# Glycolysis inhibition partially resets epilepsy-induced alterations in the dorsal hippocampus-basolateral amygdala circuit involved in anxiety-like behavior

**DOI:** 10.1038/s41598-023-33710-1

**Published:** 2023-04-21

**Authors:** Vahid Ahli Khatibi, Morteza Salimi, Mona Rahdar, Mahmoud Rezaei, Milad Nazari, Samaneh Dehghan, Shima Davoudi, Mohammad Reza Raoufy, Javad Mirnajafi-Zadeh, Mohammad Javan, Narges Hosseinmardi, Gila Behzadi, Mahyar Janahmadi

**Affiliations:** 1 Department of Physiology, School of Medicine, Shahid Beheshti University of Medical Sciences, Tehran, Iran; 2 Neurophysiology Research Center, School of Medicine, Shahid Beheshti University of Medical Sciences, Tehran, Iran; 3 Department of Physiology, School of Medicine, Tarbiat Modares University, Tehran, Iran; 4 Department of Molecular Biology and Genetics, Aarhus University, Åarhus, Denmark; 5 Stem Cell and Regenerative Medicine Research Center, Iran University of Medical Sciences, Tehran, Iran; 6 Neuroscience Research Center and Department of Physiology, School of Medicine, Shahid Beheshti University of Medical Sciences, Tehran, Iran

**Keywords:** Developmental biology, Neuroscience, Physiology

## Abstract

Pharmacoresistant temporal lobe epilepsy affects millions of people around the world with uncontrolled seizures and comorbidities, like anxiety, being the most problematic aspects calling for novel therapies. The intrahippocampal kainic acid model of temporal lobe epilepsy is an appropriate rodent model to evaluate the effects of novel interventions, including glycolysis inhibition, on epilepsy-induced alterations. Here, we investigated kainic acid-induced changes in the dorsal hippocampus (dHPC) and basolateral amygdala (BLA) circuit and the efficiency of a glycolysis inhibitor, 2-deoxy D-glucose (2-DG), in resetting such alterations using simultaneous local field potentials (LFP) recording and elevated zero-maze test. dHPC theta and gamma powers were lower in epileptic groups, both in the baseline and anxiogenic conditions. BLA theta power was higher in baseline condition while it was lower in anxiogenic condition in epileptic animals and 2-DG could reverse it. dHPC-BLA coherence was altered only in anxiogenic condition and 2-DG could reverse it only in gamma frequency. This coherence was significantly correlated with the time in which the animals exposed themselves to the anxiogenic condition. Further, theta-gamma phase-locking was lower in epileptic groups in the dHPC-BLA circuit and 2-DG could considerably increase it.

## Introduction

Epilepsy is one of the most complicated neural diseases with a high incidence among the human population^1,2^. It is characterized by increased neuronal excitability and appears as seizures; moreover, its many comorbidities, including anxiety, have a deep impact on the patients’ personal and social lives. Epilepsy has no certain treatment and the seizures and comorbidities are not controlled effectively in patients with pharmacoresistant epilepsies like temporal lobe epilepsy^3^.

Temporal lobe epilepsy has the highest rate of affliction among other focal epilepsies, and it poses a strong resistance to clinically available medications. Moreover, the current drugs have severe side effects, decreasing life quality even further. Hence, finding competent and safe therapies is of great importance^4^.

Although the effects of altered metabolism in epileptogenesis have been investigated^5,6^ the fundamental role of metabolism in regulating neuronal excitability and network circuit activity is not well determined. Metabolic manipulations like glycolysis and lactate dehydrogenase inhibition, which leads to severe energy deprivation of neurons, have been effective in controlling seizures and comorbidities in many animals studies^7–14^. Inhibition of metabolism by glycolysis inhibitors like 2-deoxy d-glucose (2-DG) has attracted attention not only because they are effective but also because they are rather safe and well tolerated^15^; indeed, 2-DG has had promising results in attenuating cancerous cell growth in clinical trials^16^, and is in clinical use to treat Covid-19^17^.

Many animal models of epilepsy have been used to evaluate the efficacy of novel therapies; among which, the intrahippocampal kainic acid model of temporal lobe epilepsy is believed to be a proper human temporal lobe epilepsy simulator because of the instantaneous seizures and hippocampal sclerosis seen in this model^18,19^. Hippocampal damage may lead to changes in cognitive function, including anxiety. Moreover, the model reveals a high level of resistance to the available medications^20^.

Dorsal hippocampus (dHPC) plays key roles in cognitive behaviors, including anxiety-like behavior; even though many lesion studies in rodents have shown that ventral hippocampus (vHPC) is more important in anxiety-like behavior compared to dHPC^21,22^, dHPC inhibition alters elevated plus maze parameters^23^. Emerging evidence shows that dHPC is involved in the acquisition of anxiety-like behaviors through various mechanisms including its connectivity to several brain regions such as the raphe nucleus and amygdala; potentiation of serotonergic inputs from the raphe nucleus to dHPC leads to increased anxiety-like behavior^24,25^. Besides hippocampus, amygdala is another key region for mediating anxiety-like behavior^26,27^. It is the first structure which receives emotional information from sensory cortices, integrates and relays this information to the medial prefrontal cortex. The hippocampus mediates this connection between the amygdala and prefrontal cortex^28^. The basolateral nucleus of the amygdala (BLA) is the main sub-region of the amygdala that is involved in anxiety-like behavior^29^.

Here, we used local field potential (LFP) recording while the animals were performing elevated zero-maze and as well as baseline condition to examine the changes in the dorsal hippocampus-basolateral amygdala neural circuit in anxiety-like behavior in the intrahippocampal kainic acid model of temporal lobe epilepsy, and following inhibition of glycolysis.

LFP recording provides us with the electrical activity in neuronal populations near the recording electrode. The signals obtained from different regions, then, can be analyzed simultaneously to reveal the connection between these structures. When the animals are doing a behavioral test, LFP recording can offer important information about how brain regions and their electrical connectivity are translated into behavioral functioning.

We hypothesized that dHPC and BLA contribute to anxiety-like behavior and their connectivity may be altered when the animals are anxious compared to the baseline condition. Moreover, we assumed that due to the severe alterations in dHPC following kainic acid injection, electrophysiological properties of dHPC and BLA, as well as dHPC-BLA connectivity, are altered in this epileptic model when the animals are exposed to anxiogenic condition and these electrical alterations are correlated to the anxiety-like behavior. We also attempted to assess the effects of 2-DG, a glycolysis inhibitor, on these possible alterations in epileptic animals. As we very recently reported that the locomotion status of mice interferes with all zero-maze parameters except for body stretching frequency^30^, which is the most emotionally driven behavior in the zero-maze test^31^, here we defined the time in which the animals were body stretching as an anxiogenic condition and compared to the baseline condition.

The present study, hence, aimed to investigate if dHPC-BLA circuit is a role player in anxiety-like behavior in rodents, as well as how the circuit is affected by epilepsy induction. Moreover, we attempted to evaluate the effect of glycolysis inhibition, a candidate as a novel antiepileptic intervention, on epilepsy-induced alterations.

## Materials and methods

### Animals

This study was carried out on 18 adult male *NMRI* mice (30 to 35 g of weight; Pasteur institute, Tehran). The animals were housed with free access to a standard pellet diet and tap drinking water ad libitum. They were kept in a temperature-controlled (23 ± 2 °C) animal house free from any source of chemical or noise pollution under the 12:12 h light: dark cycle. All animals received human care and gentle handling throughout the study, as it has been shown that proper techniques and frequency of handling were used to reduce stress and anxiety^32^. The mice were single housed after the surgery; although social housing is deemed to be the optimal way of housing, previous studies showed that single housing does not significantly affect behavioral tests in mice^33^. Hence, we single-housed the mice to prevent possible electrode dislocation as mice tend to remove each other’s cement fixed on their skull. All experimental procedures and animal care conformed to the guidelines of ARRIVE and National Institute of Health Guide for the Care and Use of Laboratory Animals and were approved by the Biomedical Research Ethics Committee of the National Institute for Medical Research Development (Approval ID: IR.NIMAD.REC.1399.259) and the Ethics Committee of Shahid Beheshti University of Medical Sciences (Authorization code: IR.SBMU.MSP.REC.1400.630).

### Study design

**Figure 1:**
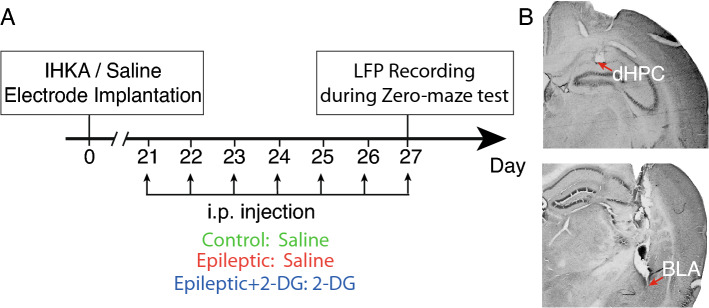
Timeline and verification of electrode sites. (**A**) A timeline depicting the study time-line; IHKA: intrahippocampal kainic acid injection. (**B**) Verification of electrode sites: dHPC: dorsal hippocampus; BLA: basolateral nucleus of the amygdala.

This study intended to assess electrophysiological impact of epilepsy induction by kainic acid injection into dHPC on the neural circuit between dHPC and BLA, which are role players in anxiety-like behavior, and how dHPC-BLA circuit is altered when the animals are exposed to the anxiogenic condition. Three separate groups were used: control group received intrahippocampal plus i.p. saline; the epileptic group received intrahippocampal kainic acid (see below) plus i.p saline; epileptic + 2-DG group received intrahippocampal kainic acid and i.p 2-DG. Intrahippocampal kainic acid or saline were injected on the 0th day; i.p saline or 2-DG were injected from 21st to the 27th days, once a day. 300 mg/kg 2-DG was dissolved in saline and injected with a volume of 0.1 mL/10 g of body weight. This dose was chosen according to the previous studies^12,13^. Saline was injected with the same volume (Fig. 1A). On the day of the experiment (27th day), 2-DG or saline were injected 90 min prior to simultaneous zero-maze test and LFP recording, a time in which 2-DG induced ketosis is noted^34^.

### Surgery, epilepsy induction and electrodes implantation

**Figure 2:**
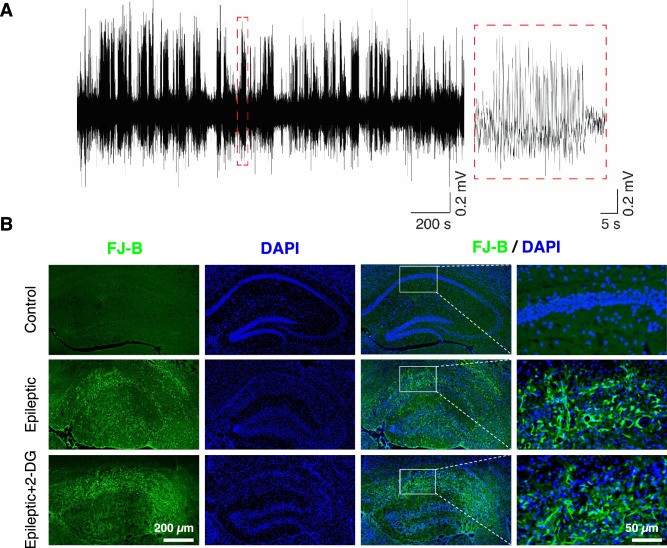
Interictal epileptiform activity and neuronal loss and degeneration in the epileptic animals. (**A**) Interictal epileptiform activity in the epileptic animals in a 30 min epoch. (**B**) The dorsal hippocampus in control (upper), epileptic (middle), and epileptic + 2-DG (lower) groups. Note severe cell loss (DAPI) and degenerating neurons (FJB) in epileptic animals. In the right column, images represent superimposed DAPI with FJB staining.

As shown previously, intrahippocampal kainic acid injection induces temporal lobe epilepsy in rodents^7,30^. Briefly, mice underwent stereotaxic surgery. They were anesthetized with an intraperitoneal injection of ketamine (100 mg/kg) and xylazine (10 mg/kg). The ear bars were placed delicately prior to muzzle fixation. Lidocaine 2% was injected under the scalp skin 5 min before making an approximately 2 cm incision in the skin. Following Bregma-Lambda adjustment to a plane level, three holes were made by a fine drill. 0.8 nmol kainic acid was dissolved in 40 nL normal saline and directly injected into the left dorsal hippocampus (−1.6 mm to the Bregma, 1.6 from the midline, and 1.2 mm deep from the dura mater) according to the directions of Paxinos and Franklin atlas (2001). To prepare electrodes, 2 stainless steel wires (127 μm in diameter, A.M. system Inc., USA) were intertwined to give the electrode suitable strength and flexibility. The electrodes, then, were soldered to a connector and placed in dHPC (−2.1 mm AP, 1.5 mm ML, 1.2 mm DV) and BLA (−1.4 mm AP, 2.5 mm ML, 3.7 mm DV). 6 screws (one as the reference electrode above the cerebellum) were screwed to the scalp. Lastly, dental cement was used to fix the electrodes. As status epilepticus following intrahippocampal kainic acid is non-convulsive, verification of the model induction was confirmed by frequent interictal epileptiform activity (see below for details) as well as severe cell loss and neuronal degeneration in the dorsal CA1 (Fig. 2 A&B).

### Elevated zero-maze test

Anxiety-like behavior was evaluated by using zero-maze apparatus^35^. The apparatus (60 cm in diameter, 5 cm wide circular corridor, 16 cm high walls and 60 cm high from the floor) was made of wood and painted black. After 30 min of habituation to the experiment room, each animal was placed in an intersection between open and closed arm facing the closed arm. During the following 5 min, the animals were videotaped then analyzed offline to find the duration of body stretching. Behavioral and electrical recordings were synchronized by an LED and body stretching times were extracted and analyzed.

### Local field potentials recordings

The implanted electrodes plugged into a miniature buffer headstage with a high-input impedance (BIODAC-A, TRITA WaveGram Co., Tehran, Iran). The headstage was connected to a main AC coupled amplifier (1000 × amplification) and to the recording system (BIODAC-ESR18622, TRITA WaveGram Co., Tehran, Iran). The LFP signals were recorded for one hour at a 1 kHz sample rate and low-pass filtered at 250 Hz while the animals were freely moving. Interictal epileptiform discharges were defined as sharp-waves, having more than twofold amplitude compared with baseline, as well as having a frequency between 1 to 20 Hz. The discharges were detected and analyzed by MATLAB software (MathWorks, Inc. version 2016a. http://www.mathworks.com). Frequent interictal discharges endorsed epilepsy induction. At the end of the experiments, brains were removed to verify the proper placement of the electrodes (Fig. 1B).

### Local field potential analyses

#### Power spectrum density and coherence

Theta (4–12 Hz) and gamma (30–50 Hz) oscillations were identified by band-pass filtering the raw LFP signals. To compute power spectral density (PSD), Welch’s periodogram function of MATLAB (MathWorks, Inc. version 2016a. http://www.mathworks.com) was utilized. We also used coherency spectra to identify the dynamic functional connectivity between different brain regions and measure the similarities between signals in the frequency domain. For this, the e coherence spectra of the dHPC-BLA circuit were analyzed by calculating magnitude-squared coherence using the “mscohere” function in MATLAB. Both power and coherence spectra computing carried 1 s hamming windows with a 90% overlap on 60 s segments of resting state as well as signals during body stretching in zero maze. In order to obtain accurate magnitudes of all the frequency components of the signals, a hamming window filter was used to reduce spectral leakage. Power spectrum density (PSD) measures a signal’s power content versus frequency while coherence is used to determine if brain regions have similar neuronal oscillatory activity across frequency steps in theta and gamma bands.

### Theta-gamma phase-phase locking

Various types of cross-frequency coupling give the notion that brain regions may exert diverse coding strategies to transform complicated information^36^. Theta-gamma phase-locking analysis has been demonstrated to take part in information routing in which multiple gamma cycles are consistently entrained during one cycle of theta^37^. Briefly, Hilbert transform of the filtered signals was used to obtain the phase of LFP signals corresponding to both theta and gamma frequency bands. The obtained phases, then, were binned into 120 bins (3-degrees width per bin), and then their two-dimensional histogram matrix was calculated, where its (*i.j*)*th* element was the proportion of the number of samples, while the theta phase was located in *ith* bin and the gamma phase was in *jth* bin, simultaneously. To smoothen the histogram, a Gaussian kernel with a standard deviation of 3 and a size of 30 bins was used. For ‘Original’ in Fig. 6A, we used the same time window to obtain theta and gamma then ‘Time Shift’ procedure was created to surrogate epochs for investigating the reliability of surrogate methods for detecting n:m phase-locking.

### Simultaneous zero-maze test and local field potential recording

Here, we sought to reveal how brain oscillations were affected by facing an anxiogenic condition compared to a baseline condition in healthy and epileptic mice. Moreover, how 2-DG can affect kainic acid-induced changes? With this regard, we extracted the time in which the mice were doing body stretching posture; they lengthen their body on the corridor to explore the open arms while a part of their body is still in the closed arm. This posture is considered to be the time in which the animal is anxious most^31^, hence, we defined it as an anxiogenic condition. the LFP parameters were calculated in this anxiogenic condition and compared to the values extracted from the baseline condition; the baseline condition was a 120 s epoch in which the animals were immobile but not asleep.

### Detection of degenerating neurons and apoptosis by Fluoro-Jade B and DAPI staining

To detect degenerating neurons in kainic acid-treated dorsal hippocampi, Fluoro-Jade B staining was performed as described before^38^; the mice were anesthetized (100 mg/kg ketamine and 10 mg/kg xylazine, i.p) on the day 27 and perfused transcardially with a cold fixative containing 4% paraformaldehyde and 1.33% picric acid in 0.1 M phosphate buffer (PB, pH 7.4) following 0.9% saline perfusion. The brains were then dissected out from the skull, post-fixed overnight in the same fixative at 4 °C and cryoprotected by being immersed in 20% sucrose until they sank. The brains were freeze-sectioned coronally at 10 µm thickness, between the AP 1.2 mm and 2.4 mm posterior to the Bregma (Paxinos & Franklin, 2001) using a cryostat (Leica CM1850, Germany). The slides were first immersed in 1% sodium hydroxide in 80% ethanol for 5 min; this was followed by 2 min in ethanol 70% prior to 10 min in 0.06% potassium permanganate solution. The slides, then, were immersed in 0.0004% Fluoro-Jade B (FJB) solution for 20 min and rinsed with distilled water afterwards. To detect apoptosis, the slides were counterstained with DAPI staining solution, and then washed with distilled water, air dried, cleared, and coverslipped. Imaging was performed using a fluorescent microscope (Olympus, BX51 TRF, USA) equipped with a DP72 CCD camera (Olympus, Japan). ImageJ software (National Institutes of Health, Bethesda, Maryland, USA. http://imagej.nih.gov/ij) was used to prepare the images.

## Results

### Intrahippocampal kainic acid led to ubiquitous interictal epileptiform activity and severe cell loss as well as ongoing neuronal degeneration in dHPC

First, we sought to confirm the model induction by assessing 1-h continuous LFP recording and cell loss in the dorsal hippocampus. As it is illustrated in Fig. 2A, interictal epileptiform discharges were frequently seen during LFP accompanied by severe cell loss and ongoing neuronal degeneration in dorsal CA1 of kainic acid-treated animals (Fig. 2B). 2-DG could not exert any histological beneficial effects as it was injected long after kainic acid injection (see above). Hence, intrahippocampal kainic acid injection leads to frequent interictal epileptiform activity and severe cell loss, as well as neurons undergoing degeneration in dorsal CA1.

### Epilepsy induction disrupted theta and gamma powers

**Figure 3:**
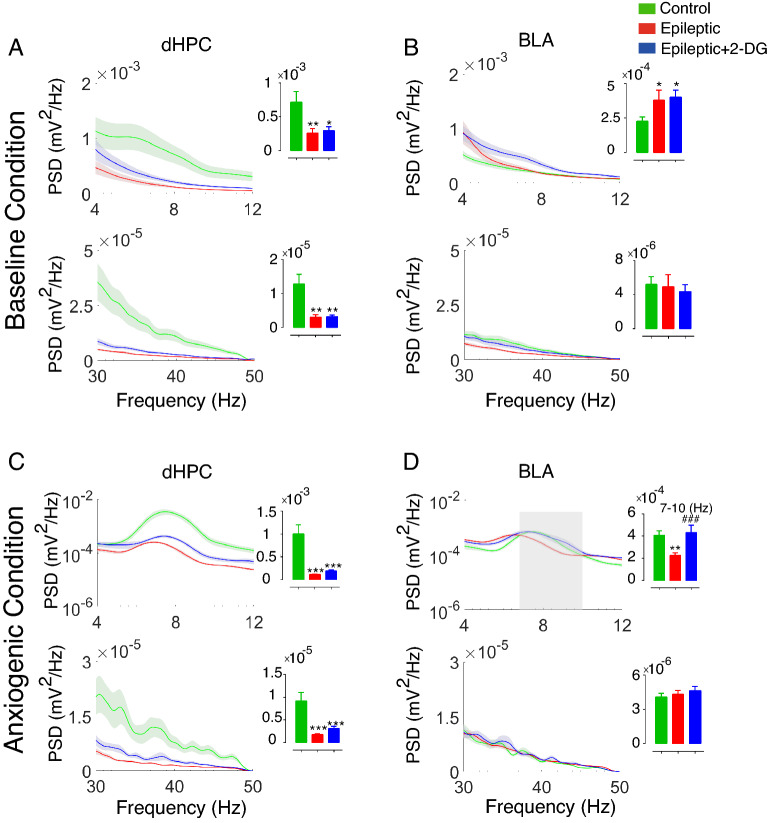
Power spectrum density in dHPC and BLA during baseline and anxiety. (**A**) A decreased theta (4–12 Hz) and gamma (30–50 Hz) powers was observed in dHPC of the epileptic animals compared to control in the baseline condition. (**B**) Theta power in BLA in epileptic and epileptic + 2-DG groups increased compared to the control group in baseline condition while gamma power was not significantly different between the groups. (**C**) During anxiety, theta and gamma powers in dHPC in epileptic and epileptic + 2-DG groups decreased compared to control groups. (**D**) Theta power in BLA was lower in epileptic animals while 2-DG could completely reverse this decrement. The data were compared using one-way ANOVA and are shown as mean ± SEM. *p < 0.05, **p < 0.01, ***p < 0.001 between control and epileptic, control and epileptic + 2-DG groups. ###p < 0.001 between epileptic and epileptic + 2-DG groups. dHPC: dorsal hippocampus; BLA: basolateral nucleus of the amygdala.

To assess the alterations of oscillatory activity following epilepsy induction, we evaluated PSD in dHPC during both baseline and anxiogenic conditions. Theta and gamma powers were significantly lower in the dorsal hippocampus of the epileptic animals meanwhile 2-DG failed to reverse decreased power in epileptic animals (Fig. 3A&C).

Further, theta power of BLA during baseline condition increased in epileptic and epileptic + 2-DG groups compared to control animals (Fig. 3B); in anxiogenic condition (the time during which the mice were stretching their body from closed arms to open arms), however, high theta power was significantly lower in the epileptic group compared to control and epileptic + 2-DG groups (Fig. 3D). Nonetheless, BLA gamma power was not notably different between the groups, both in baseline and anxiogenic conditions (Fig. 3B&D). PSD assessment, therefore, indicates that dHPC powers decrease both in theta and gamma frequency bands in baseline and anxiogenic conditions in epileptic animals while BLA theta power is higher in epileptic animals only when they are immobile; in anxiogenic condition, BLA theta power decreases in epileptic animals and 2-DG can reverse it considerably.

### Epilepsy induction altered coherence in dHPC-BLA circuit in anxiogenic condition

**Figure 4:**
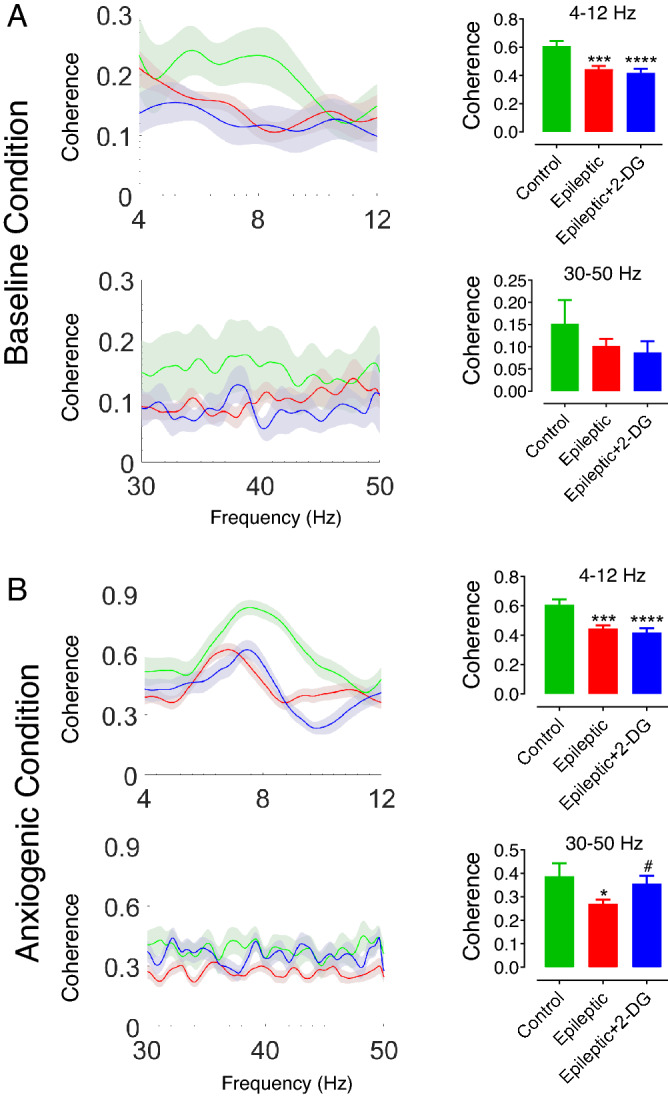
Coherence between in dHPC-BLA circuit during the baseline and anxiogenic conditions. Theta (4–12 Hz) and gamma (30–50 Hz) coherence between dHPC and BLA was not significantly different between the groups in the baseline condition (**A**). In anxiogenic condition, theta coherence was significantly lower in epileptic animals; gamma coherence was significantly lower in epileptic animals compared to control values while 2-DG significantly increased it in epileptic + 2-DG group compared to epileptic group (**B**). The data were compared using one-way ANOVA and are shown as mean ± SEM. *p < 0.05, ***p < 0.001 between control and epileptic, control and epileptic + 2-DG groups. #p < 0.05 between epileptic and epileptic + 2-DG groups. dHPC: dorsal hippocampus; BLA: basolateral nucleus of the amygdala.

Having noted the altered power values in epileptic animals both in dHPC and BLA, we hypothesized that the connectivity between these two structures had changed. Our assessment of coherence in the dHPC-BLA circuit revealed no significant difference among the groups during the baseline condition (Fig. 4A). However, in the anxiogenic condition in zero-maze apparatus, amazingly, theta coherence between dHPC and BLA was significantly higher in control groups compared to epileptic animals. 2-DG could not reset the decreased coherence in epileptic animals at theta frequency band. Likewise, coherence at the gamma band was significantly lower in epileptic animals while a rise was found in the epileptic + 2-DG group compared to the epileptic group (Fig. 4B). As a conclusion, the difference in coherence values between the groups was evident only when the animals were exposed to the anxiogenic condition. Only in the case of the gamma band, 2-DG could reverse decreased coherence in epileptic animals.

### Coherence in dHPC-BLA circuit is correlated to anxiogenic condition

**Figure 5:**
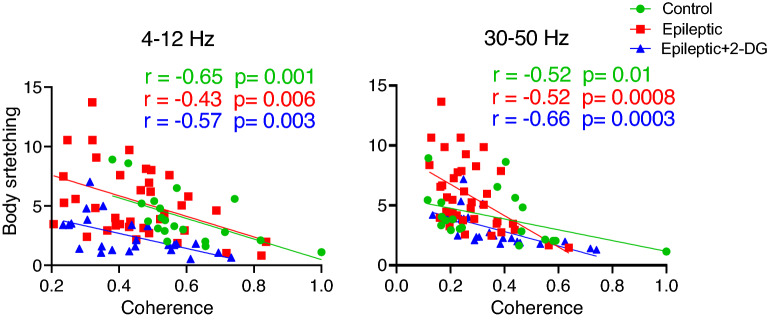
Correlation of anxiogenic condition with BLA-dHPC coherence. Coherence between dHPC-BLA at theta frequency (left) as well as gamma frequency (right) is negatively correlated with the anxiogenic condition (body stretching duration). Pearson correlation coefficients were used to evaluate the correlation. dHPC: dorsal hippocampus; BLA: basolateral nucleus of the amygdala.

As epileptic induction disrupted the connection between dHPC and BLA, we further evaluated its correlation with the anxiogenic condition. Our findings illustrated that the coherence values between dHPC and BLA at both theta and gamma frequencies were negatively correlated with the time in which animals exposed themselves to the anxiogenic condition in all three groups (Fig. 5). Moreover, the absolute value of the correlation in the theta band, although not significantly, was reduced in epileptic animals, while 2DG led to an increase in the correlation compared to the epileptic group. However, no noticeable changes in the correlation between coherence in the dHPC-BLA circuit with the anxiogenic condition were noted in the gamma band between the control and epileptic group; this correlation in the gamma band, however, was slightly elevated in the epileptic + 2-DG group. Therefore, coherence in the dHPC-BLA circuit is correlated to the spent time in the anxiogenic condition in the control and the epileptic group, even though it is weaker in epileptic animals. 2-DG reversed this weakened correlation.

### Phase-phase coupling between dHPC and BLA was different in baseline and anxiogenic conditions

**Figure 6:**
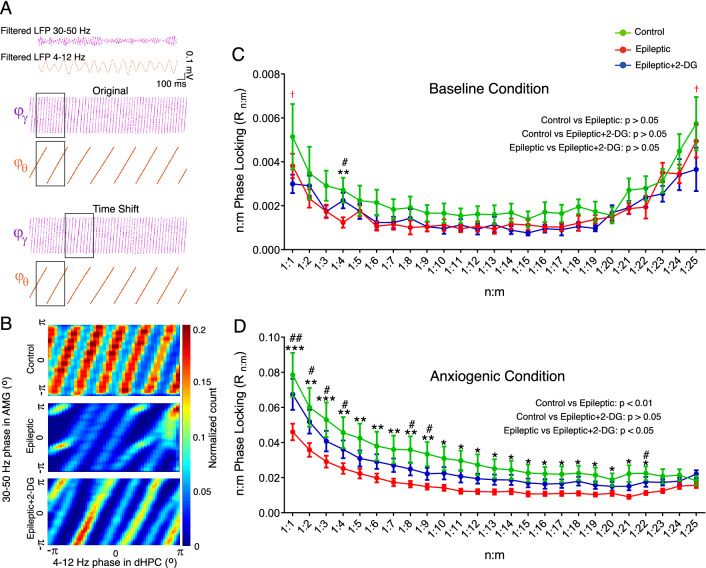
Phase-phase coupling between the dorsal hippocampus and basolateral nucleus of the amygdala. (**A**) The top panel shows filtered LFP data at theta (4–12 Hz, orange) and gamma (30–50 Hz, purple) frequencies. The bottom panels illustrate 500 ms of the instantaneous phase-time series in the two signals. The Phase-Locking Index (n:m) was computed to estimate the consistency of the phase difference between accelerated theta and gamma oscillations in original and time shifted window. (**B**) 2D phase-phase histogram samples in the three groups. The X-axis depicts a period of dHPC theta oscillation during which BLA gamma oscillation periods are shown (Y axis). R (n:m) which shows the theta and gamma phase difference in various time shifts was evaluated for each proportion of theta/gamma in baseline (**C**) and anxiogenic (D) conditions (for instance, 1:5 means that dHPC theta is accelerated 5 times to assess the consistency of its phase with the phase of BLA gamma oscillation). The data were compared using two-way ANOVA and are shown as mean ± SEM. *p < 0.05, **p < 0.01, ***p < 0.001 for comparison between control and epileptic groups. ^†^p < 0.05 to compare control with epileptic + 2-DG group. #p < 0.05, ##p < 0.01to compare epileptic and epileptic + 2-DG groups. dHPC: dorsal hippocampus; BLA: basolateral nucleus of the amygdala.

We finally investigated altered functional connectivity between dHPC and BLA using phase-phase coupling analysis. First, our findings revealed that theta-gamma phase-locking (BLA gamma phase locked to dHPC theta phase) was disrupted in epileptic animals (Fig. 6B, see below). Moreover, in baseline condition, the phase-locking values were significantly lower in epileptic animals compared to controls only in 1:1, 1:4 and 1:25. 2-DG significantly reversed the alteration in only 1:4 (Fig. 6C). In the anxiogenic condition, however, in the points from 1:1 to 1:22, phase-locking was much stronger in control animals compared to the epileptic group. Interestingly, 2-DG could substantially increase phase-locking in many points (Fig. 6D). Hence, the dHPC theta phase regulates the BLA gamma phase both in baseline and anxiogenic conditions, but it is much stronger in the latter, indicating elevated dHPC-BLA connectivity in anxiety. The phase-locking was weaker in epileptic animals and 2-DG could reset it at many points.

### Coherence and phase-locking values in the dHPC-BLA circuit were much higher in anxiogenic condition

Apart from changes induced by epilepsy induction we reported, the differences in the LFP parameters within each group in anxiogenic condition compared to baseline could indicate the importance of dHPC-BLA circuit in anxiety-like behavior. Interestingly, in the anxiogenic condition, theta and gamma powers were significantly lower in the epileptic group, but not in control mice, compared to baseline. However, 2-DG could reverse the alterations induced by kainic acid treatment. dHPC-BLA circuit coherence and phase-locking, however, were substantially higher when the animals were in anxiogenic conditions compared to the baseline in all the experimental groups. This may suggest the importance of the dHPC-BLA circuit in anxiety-like behavior in mice (Table 1).

**Table 1:** Comparison of power, coherence and phase-locking between baseline and anxiogenic conditions within each experimental group.

	Baseline condition		
	Control	Epileptic	Epileptic + 2-DG
Anxiogenic condition			
dHPC Theta Power	0.0002 ± 0.0003	−0.0001 ± 3.794e−005***	−0.0001 ± 5.362e−005
BLA Theta Power	2.455e−005 ± 4.020e−005	−0.0001 ± 5.321e−005*	−9.061e−005 ± 6.859e−005
dHPC Gamma Power	−3.636e−006 ± 3.333e−006	−1.244e−006 ± 4.910e−007**	−1.146e−007 ± 9.108e−007
BLA Gamma Power	−1.103e−006 ± 7.878e−007	−5.858e−007 ± 9.712e−007	2.660e−007 ± 7.816e−007
Theta Coherence	0.41 ± 0.05***	0.30 ± 0.05***	0.29 ± 0.05***
Gamma Coherence	0.23 ± 0.08**	0.16 ± 0.04***	0.26 ± 0.06***
Phase-locking	0.03 ± 008**	0.01 ± 0.003***	0.02 ± 0.005***

dHPC theta and gamma powers as well as BLA theta power were lower in anxiogenic condition only in epileptic group and 2-DG could reverse the decreased powers. Theta and gamma coherence as well as phase-locking between dHPC and BLA were substantially higher in anxiogenic condition compared to baseline in all the three groups. Unpaired t-test was used to compare the values. The data are shown as the difference between means (anxiogenic values – baseline values) ± SEM. B: anxiogenic condition; A: baseline condition. *p < 0.05, **p < 0.01, ***p < 0.001.

## Discussion

In the present study, we set out to address the electrophysiological signature of dHPC-BLA circuit in anxiety-like behavior in intact and epileptic mice. Moreover, we evaluated the effects of glycolysis inhibition on epilepsy-induced changes. Our findings from simultaneous LPF recording during anxiety-like behavior and histochemical staining indicate that intrahippocampal kainic acid injection leads to severe cell loss as well as generating frequent interictal epileptiform activity in the CA1 pyramidal cell layer endorsing epilepsy induction. Moreover, even 27 days after kainic acid injection, many neurons undergo a degeneration process. This severe cell loss and degeneration bring about profound alterations in the electrical properties of kainic acid injected dHPC as well as intact BLA. Moreover, the dHPC-BLA circuit is disrupted in epileptic animals, as was evidenced by decreased coherence in the circuit. This decrease in coherence is negatively correlated to the time in which mice expose themselves to anxiogenic conditions (body stretching posture). Furthermore, weakened theta-gamma phase-locking between dHPC and BLA is noted in the epileptic animals. Most interestingly, the alterations following epilepsy induction are obvious only when the animals are exposed to the anxiogenic condition compared to baseline condition; this is true, especially in the case of coherence and phase-locking assessments. Moreover, the connectivity between dHPC and BLA is substantially higher when the animals are anxious regardless of the groups indicating the importance of dHPC-BLA circuit in anxiety-like behavior.

Decreased theta power, which is the dominant brain oscillation in the hippocampus, has been reported in the intrahippocampal kainic acid model of temporal lobe epilepsy^39^. We have also very recently reported that intrahippocampal kainic acid injection leads to severe cellular and electrophysiological alterations in dHPC^30^.

Unlike BLA and vHPC which are directly interconnected, dHPC is not directly connected to BLA; rather, the connection between the two structures has been reported to be mediated by lateral entorhinal and perirhinal cortices; the proposed circuit involves outputs from dorsal CA1/subiculum to entorhinal/perirhinal cortices, which are robustly connected to amygdala^40^. BLA-vHPC circuit is important in regulating anxiety^41^. Likewise, dHPC-BLA hyperconnectivity was reported in rodents with chronic exposure to anxiogenic conditions in the early stages of life^41^. Moreover, injection of both the serotonin receptor (5-HT_6_) agonist and antagonist into dHPC exerts anxiolytic effects; moreover, amygdala dopamine level is altered by changes in serotonin signaling, which plays an important role in anxiety^25,43^. Most of the previous studies have used lesions or pharmacological interventions to inhibit or stimulate the brain regions and then performed behavioral experiments to reveal how the inhibition or stimulation affects behavioral functioning.

Glycolysis inhibition by 2-DG can reverse many alterations induced by epilepsy induction including gamma coherence and theta-gamma phase-locking in dHPC-BLA circuit. Further, it increases BLA theta power when the epileptic animals are exposed to anxiogenic condition.

The decreased theta power in dHPC we reported here, both in baseline and anxiogenic conditions, is consistent with previous findings showing attenuated theta oscillations in kainic acid-treated CA1^39^. Both hippocampal pyramidal neurons and interneurons have been reported to contribute to the generation of hippocampal theta oscillations; indeed, hippocampal theta rhythm is generated by the medial septum, nucleus incertus and entorhinal cortex and pyramidal neurons follow this rhythm^44^; in turn, the pyramidal neurons project back to the medial septum and help the maintenance of the theta rhythm^45,46^. The GABAergic interneurons play a crucial role in generating such coordination between the pyramidal neurons and theta generators^47–49^. Hence, such severe cell loss and ongoing degeneration in CA1 would lead to such decrement in theta power we noted. Hippocampal gamma oscillations, however, are generated more locally. Two possible mechanisms have been explored regarding whether special synaptic properties at the gamma generating loci are the main players or neuromodulation (cholinergic for instance) plays the main role in generation of hippocampal gamma oscillations. Regardless of which mechanism is more important, both emphasize on the interactions between pyramidal and interneurons^50,51^, which are disrupted in the kainic acid-treated hippocampus as was evidenced by severe cell loss. Therefore, a gamma power decrement in CA1 is expected as we demonstrated. 2-DG was unable to reverse the decreased power as it was injected long after the cell loss.

Interestingly, kainic acid-treated animals revealed higher theta power in BLA in baseline condition compared to control and 2-DG treatment could not reverse it in epileptic animals. It is already known that the kainic acid-induced lesion does not spread out of the hippocampus at low doses like ours^52,53^. Hence, alterations in dHPC are likely to affect its connection with the other parts of the limbic formation including BLA. BLA receives information from somatosensory cortices and relays them to the hippocampus; the information, then, is evaluated by the hippocampus and sent back to BLA to affect behavior in response to various emotional stimuli^40^. This interconnection between BLA and dHPC is mediated by the entorhinal cortex^54^. When the animals are in the baseline condition, various somatosensory stimuli are sent to BLA and the dHPC to be evaluated; it is possible that due to attenuation of dHPC outputs to BLA following epilepsy induction, BLA theta power increases, as we showed here, to maintain its cooperation with dHPC. Consistently, gamma power was not affected in BLA in epileptic mice as it is known that synchronizing rhythm within the limbic formation, dHPC and BLA included, is theta, not gamma rhythm^55^. When exposed to anxiogenic condition, however, we report that BLA theta power is lower in epileptic animals and 2-DG substantially resets the power. Indeed, theta power in BLA increased in anxiogenic condition compared to baseline in control animals while it decreased in the anxiogenic condition in epileptic animals. To address such disrupted power in both BLA and dHPC we evaluated coherence in the dHPC-BLA circuit. It has been reported that the LFP power is associated with low or high-activity of the mice rather their location (open arm or open arm) in elevated plus maze test^56^. Moreover, it has been demonstrated that a short time before deciding to avoid or approach to open arm in elevated plus maze, vHPC-BLA, but not dHPC-BLA circuit plays a role in the risk assessment^57^. Here, analyzing exactly the time in which the animals were anxious most, importance of dHPC-BLA circuit emerges (see below).

In the baseline condition, no altered theta coherence was noted in the circuit between the groups. Amazingly, theta coherence between dHPC and BLA was much stronger when the animals were anxious in control animals suggesting the importance of this circuit in anxiety-like behavior. This coherence was weaker in epileptic animals and 2-DG could not strengthen it. Here we could speculate that decreased theta power in both BLA and dHPC led to such weaker coherence between them in epileptic animals when they were exposed to the anxiogenic condition. Not only is the dHPC-BLA circuit regulated by cortical input, but it may be adjusted by other subcortical areas like median raphe nucleus (MRN). MRN outputs to dHPC have been reported to regulate anxiety behavior^24,25^. interestingly, stimulation of serotonergic projections from MRN to dHPC leads to an increase in BLA dopamine which plays a role in anxiety behavior^25,43^. disrupted MRN-dHPC connection following severe cell loss and deformation of dorsal CA1, hence, may be another mechanism leading to decreased coherence between dHPC and BLA.

In the epileptic group, gamma coherence diminished in the dHPC-BLA circuit in the anxiogenic condition and 2-DG could significantly reverse it. This is interesting because BLA gamma power was not affected by epilepsy induction. decreased dHPC gamma power did not lead to gamma coherence decrement in baseline condition; hence, disrupted dHPC theta power might have led to such a decrease in gamma coherence; moreover, 2-DG could reverse it probably by increasing BLA gamma power as we showed here. Addressing this speculation begs the question whether BLA gamma activity is regulated by dHPC theta oscillations. Theta-gamma phase-locking has been shown to be a mechanism through which various regions cross-talk^37^. Moreover, medial prefrontal cortex theta phase regulates BLA firing leading to safety seeking of mice^58^. Here we analyzed theta-gamma phase-locking between the two structures. It was amazing that the coupling was much stronger when the animals were anxious suggesting that the degree to which dHPC theta regulates BLA gamma, is an important factor in anxiety-like behavior; to explain more, decreased phase-locking between dHPC and BLA may lead to decreased gamma coherence we demonstrated here; Moreover, we report that coherence in the dHPC-BLA circuit is negatively correlated to spent time in anxiogenic condition. Consequently, decreased gamma coherence resulting from attenuated theta-gamma phase-locking in the dHPC-BLA circuit leads to more spent time in the anxiogenic condition in epileptic animals compared to control values.

## Conclusion

Our data indicate that severe cell loss in dHPC leads to deep alterations in the electrophysiological properties of dHPC as well as BLA in intrahippocampal kainic acid-treated mice. Decreased dHPC theta and gamma power occur in baseline condition while BLA theta power is elevated maybe because BLA is trying to maintain its connectivity with damaged dHPC. 2-DG had no notable effects on baseline theta and gamma power. In anxiogenic condition, however, dHPC theta and gamma power reduces in epileptic animals; while, amazingly, BLA theta power decreases may be due to the fact its connectivity with dHPC is disrupted. Coherence evaluation in the dHPC-BLA circuit revealed no significant difference between control and epileptic groups in the baseline condition. Nevertheless, importantly, in the anxiogenic condition, the coherence increases dramatically illuminating the importance of the dHPC-BLA circuit in anxiety-like behavior; this is endorsed by the negative correlation between the dHPC-BLA circuit and spent time in the anxiogenic condition. This correlation, although weaker, was noted in epileptic animals as well. Having noted decreased gamma coherence in the circuit while BLA power was not affected by epilepsy induction, we set out to assess if dHPC theta regulates BLA gamma; phase-locking analysis revealed that dHPC theta highly regulates BLA gamma in the control group, especially in anxiogenic condition. Decreased phase-locking between the dHPC-BLA, hence, may lead to decreased BLA gamma power and dHPC-BLA coherence as we noted here. Hence, analyzing the time in which the animals are anxious most instead of analyzing the whole time the animals are in open or closed arm in the elevated mazes offer more precise circuit-level signature of neural circuits regarding anxiety-like behavior, as we reported here. 2-DG could reverse decreased gamma power and weakened coherence and phase-locking between dHPC and BLA. Therefore, since patients with temporal lobe epilepsy show hippocampal sclerosis (indicating cell loss), prescribing agents like 2-DG which are capable of resetting disrupted changes in limbic formation especially between the hippocampus and amygdala which play a crucial role in emotion and cognition, could exert beneficial effects.

## Data Availability

The data are available from the corresponding author on a reasonable request.
